# Glucagon-like peptide-1 receptor agonists and pancreatic cancer: a meta-analysis with trial sequential analysis

**DOI:** 10.1038/s41598-019-38956-2

**Published:** 2019-02-20

**Authors:** Lana C. Pinto, Mariana R. Falcetta, Dimitris V. Rados, Cristiane B. Leitão, Jorge L. Gross

**Affiliations:** Division of Endocrinology, Hospital de Clínicas de Porto Alegre, Universidade Federal do Rio Grande do Sul, Ramiro Barcelos St, 2350, Prédio 12, 4th floor, ZIP 90035-903 Porto Alegre, Brazil

## Abstract

We aimed to assess if GLP-1 agonists are associated with pancreatic cancer. Systematic review and meta-analysis of randomized trials with GLP-1 agonists as an intervention was performed. Trial sequential analysis (TSA) was performed to assess if the available information is sufficient to reject this association. Twelve trials met the study criteria, with a total of 36, 397 patients. GLP-1 analogues did not increase the risk for pancreatic cancer when compared to other treatments (OR 1.06; 95% CI 0.67 to 1.67; *I*^2^ 14%). TSA confirmed that enough patients were randomized and again no association of the medications and pancreatic cancer was observed considering a NNH of 1000 and the short mean follow-up of the included trials (1.7 years). Larger studies with longer duration would be required to exclude a greater NNH and to aside concerns regarding possible influence of study duration and the outcome.

## Introduction

Glucagon-like peptide-1 (GLP-1) agonists bind and activate the GLP-1 receptor, which results in lower glucose plasma values in diabetic subjects, increased satiety and reduced body weight. GLP-1 agonists promote the release of insulin in response to hyperglycaemia, inhibit the secretion of glucagon, slow gastric emptying, and augment satiety by directly affecting the central nervous system^1^. Receptors for GLP-1 are found in pancreatic islets and in pancreatic acini and ducts; basic research shows that GLP-1 therapy may lead to acinar and duct cell proliferation^2,3^.

Based on observational data, a 2011 report identified an increased risk for pancreatitis and pancreatic cancer in patients on incretin therapy^4^, which led to a Food and Drug Administration (FDA) warning on the pancreatic safety of GLP-1 agonists^5^. Two short-term studies were performed at the FDA’s request. These studies were carried out with exenatide and liraglutide in a rat model of diabetes, and they increased concerns with respect to the possible adverse effects of GLP-1 mimetic therapy on exocrine pancreas. Both drugs led to an elevation in pancreatic enzymes. One rat treated with exenatide died of pancreatic necrosis, and other animals had findings of acinar-to-ductal metaplasia and foci of ductal hyperplasia, which were interpreted as premalignant changes^6,7^.

Later, a systematic review of case reports suggested that liraglutide therapy was associated with acute pancreatitis^8^. Nonetheless, a recent meta-analysis by Storgaard *et al*.^9^, which included only trials with adjudicated pancreatitis events, did not show an association of GLP-1 agonists and acute pancreatitis^9^.

Notably, there still remains a controversy regarding pancreatic cancer. This topic was evaluated in two large cohort studies: in the first study, an increased risk for pancreatic cancer was observed in “new users”^10^, whereas no relation was observed in the second study^11^. Another recent meta-analysis reported no association between GLP-1 agonist use and pancreatic cancer^12^. However, no attempt was made to ascertain if the available number of patients on GLP-1 agonist use or the number of events were enough for definitive conclusions. In the case of a meta-analysis with a negative result, it is crucial to establish if the pooled information is sufficiently powered to exclude the association between the factor being studied (GLP-1 agonist use) and the outcome (pancreatic cancer). Since the relation between GLP-1 agonists and pancreatic cancer is still unclear, the aim of this systematic review and meta-analysis is to assess if these anti-hyperglycaemic medications have an association with pancreatic cancer. We also aimed to determine if there is sufficient evidence to exclude this association by means of trial sequential analysis (TSA).

## Methods

Preferred Reporting Items for Systematic Reviews and Meta-analyses (PRISMA) protocol recommendations’ were followed^13^ and was registered at the International Prospective Register of Systematic Reviews (PROSPERO) under the number CRD42016953346.

We searched MEDLINE, EMBASE, Cochrane Central and Clinicaltrials.gov from database inception to September 2017. The search strategy combined the Medical Subject Heading (MeSH) terms “exenatide” OR “liraglutide” OR “semaglutide” OR “dulaglutide” OR “albiglutide” OR “lixizenatide” AND “diabetes mellitus, type 2” AND a filter to identify RCTs^14^. Regardless of language, all eligible trials were considered for review.

The inclusion criteria were as follows: (1) RCTs, (2) GLP-1 agonist use versus any comparator, (3) treatment for at least 48 weeks, (4) definition of events of pancreatic cancer, (5) inclusion of patients ≥ 18 y old, and (6) diagnosis of type 2 diabetes according to the American Diabetes Association criteria^15^.

For trials that fulfilled all inclusion criteria but did not mention pancreatic cancer events, an e-mail was sent to the corresponding author asking for the data. Of 17 e-mails sent, four e-mails were returned to sender (the e-mail of the author did not exist or had changed) and 4 e-mails received replies, two of them containing pancreatic cancer data.

Two independent investigators (L.C.P. and M.R.F.) selected studies based on titles and abstracts. Studies that met the inclusion criteria, or those with abstracts that lacked information to decide upon their exclusion, were included in full-text evaluation. Both investigators also analysed full texts and extracted data.

### Risk of bias in individual studies and the quality of meta-analysis

In order to assess the quality of studies, the Cochrane Collaboration tool for risk of bias was used^16^. Regarding risk of bias, we considered the non-adjudication of events to be “other bias”. Quality of meta-analysis was evaluated by the Grading of Recommendations Assessment, Development and Evaluation (GRADE) approach^17^.

### Data Analysis

We compared the events of interest in patients randomized to use of GLP-1 agonists versus the events in patients randomized to the control strategy (placebo or other antihyperglycemic medications). The outcome of interest was pancreatic cancer.

Data were summarized with Mantel-Haenszel odds ratio (OR) with direct meta-analysis to compare the GLP-1 agonist group with the control group. Heterogeneity was assessed by the Cochran Q test (*p*-value of 0.1 was considered statistically significant) and the *I*^2^ test (values greater than 50% were considered to indicate elevated statistical heterogeneity).

We performed a TSA on the identified studies to address whether the current evidence might be sufficient for firm conclusions. This analysis is associated with a cumulative meta-analysis represented by the Z-curve. Therefore, we were able to estimate the sample size required to accept or reject a minimal difference between GLP-1 agonists and control^18,19^. This difference is arbitrary and must be clinically relevant. We set it as an absolute difference of 0.1% between groups, which is more conservative than the difference found in previous trials^20^. We conducted the TSA with an overall 5% risk of type I error and 20% risk of type II error (power of 80%). In this way, the analysis is able to reach a number needed to harm (NNH) of at least 1000. For studies with zero events in both arms, continuity correction was performed, and their data were included in TSA analyses.

Publication bias was evaluated with a visual inspection of funnel plots and with Begg’s and Egger’s tests. If a small study bias was identified, we then performed the trim and fill computation to explore the effect of missing studies on the outcomes.

The analyses were performed using RevMan software version 5.3 (Cochrane Collaboration, Copenhagen, Denmark) and STATA 12.0 (Stata Inc., College Station, Texas, USA). The TSA was performed with TSA software (Centre for Clinical Intervention Research Department, Copenhagen, Denmark).

## Results

**Figure 1:**
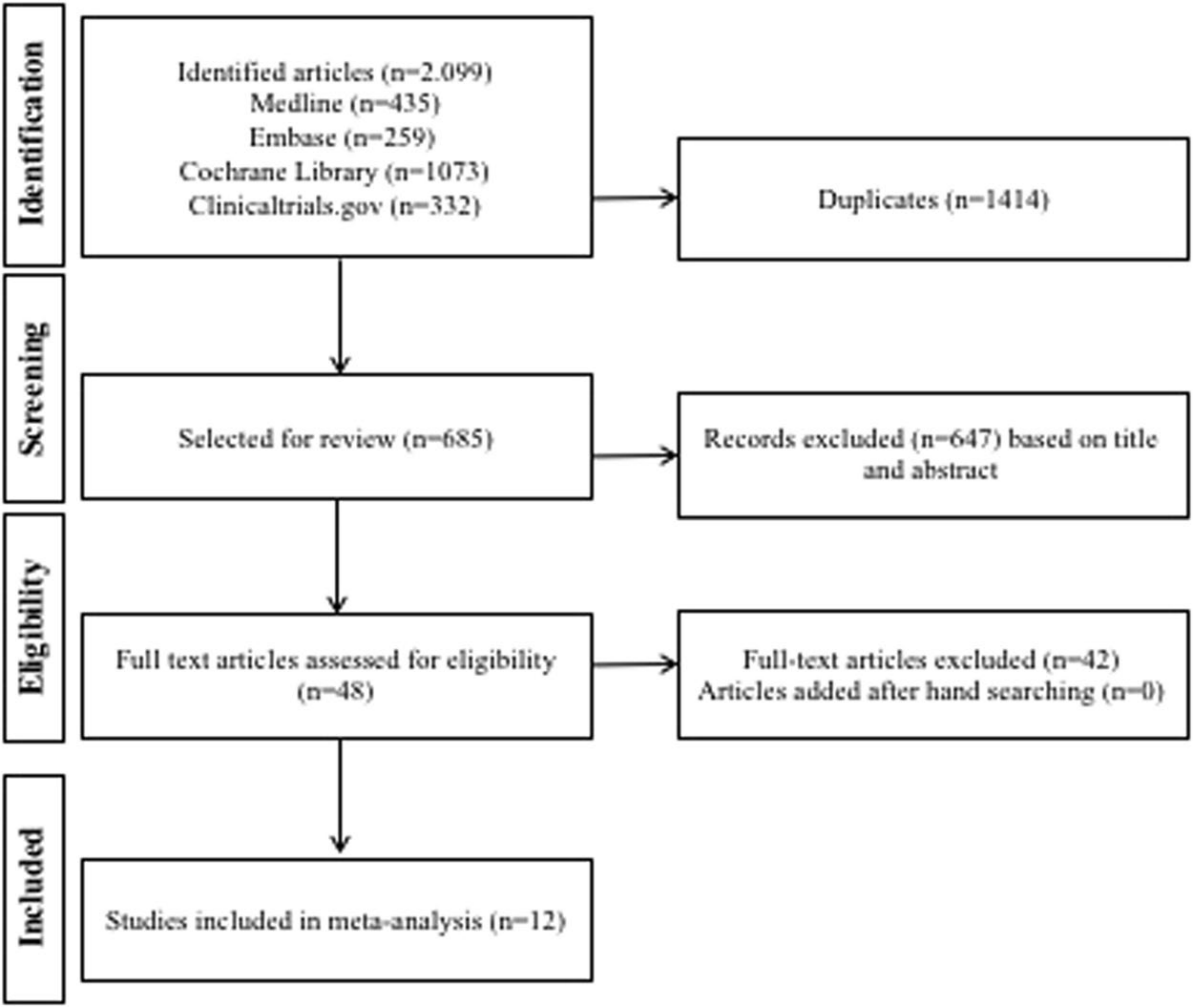
Study Flowchart.

Our search retrieved 2099 articles. After running through titles and abstracts and removing duplicates, 48 articles remained for full-text evaluation. Finally, 12 trials were included for analysis (Fig. 1). In four of these trials, pancreatic cancer events were adjudicated.

Selected studies were published between 2011 and 2017. The mean trial duration was 1.74 years. The trials included 36,397 patients, 62.77% of whom were men and with a mean age of 58.0 ± 4.3 years. Characteristics of included trials are presented in Table 1. The results regarding the individual quality of included trials are presented in Supplemental Material (Fig. 1s).

**Table 1:** Characteristics of Included Trials.

Author, Year	GLP-1 Agonist	Events Control	Events Intervention	Patients Control	Patients Intervention
**Marso**, 2016 (SUSTAIN-6)	Semaglutide	4	1	1649	1648
**Pfeffer**, 2015 (ELIXA)	Lixizenatide	9	3	3034	3034
**Marso**, 2016 (LEADER)	Liraglutide	5	13	4672	4668
**Holman**, 2017 (EXSCEL)	Exenatide 1w*	15	16	7396	7356
**Nauck**, 2016	Dulaglitide	0	1	101	200
**Kramer**, 2015	Liraglutide	0	0	25	26
**Home**, 2015	Albiglutide	1	0	277	271
**Diamant**, 2014	Exenatide 1w*	0	0	233	223
**Sathyanarayana**, 2011	Exenatide 2d*	0	0	10	11
**Pratley**, 2011	Liraglutide	0	1	219	445
**Bolli**, 2014	Lixizenatide	0	1	160	322
**Xu**, 2014	Exenatide 2d*	0	0	274	142

^*^Exenatide 1w = exenatide once weekly; exenatide 2d = exenatide twice a day.

**Figure 2:**
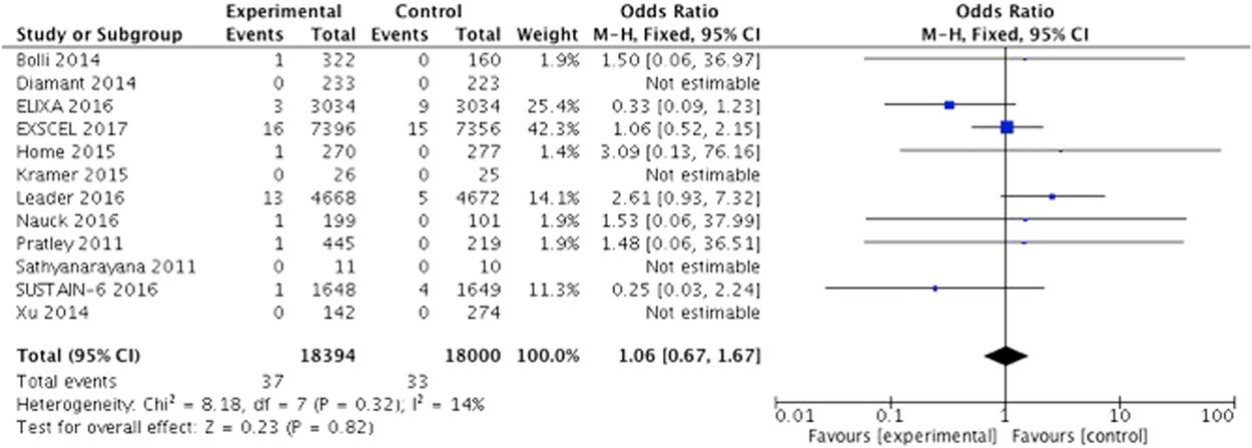
Forest Plot for pancreatic cancer in GLP1 analogs vs. control.

**Figure 3:**
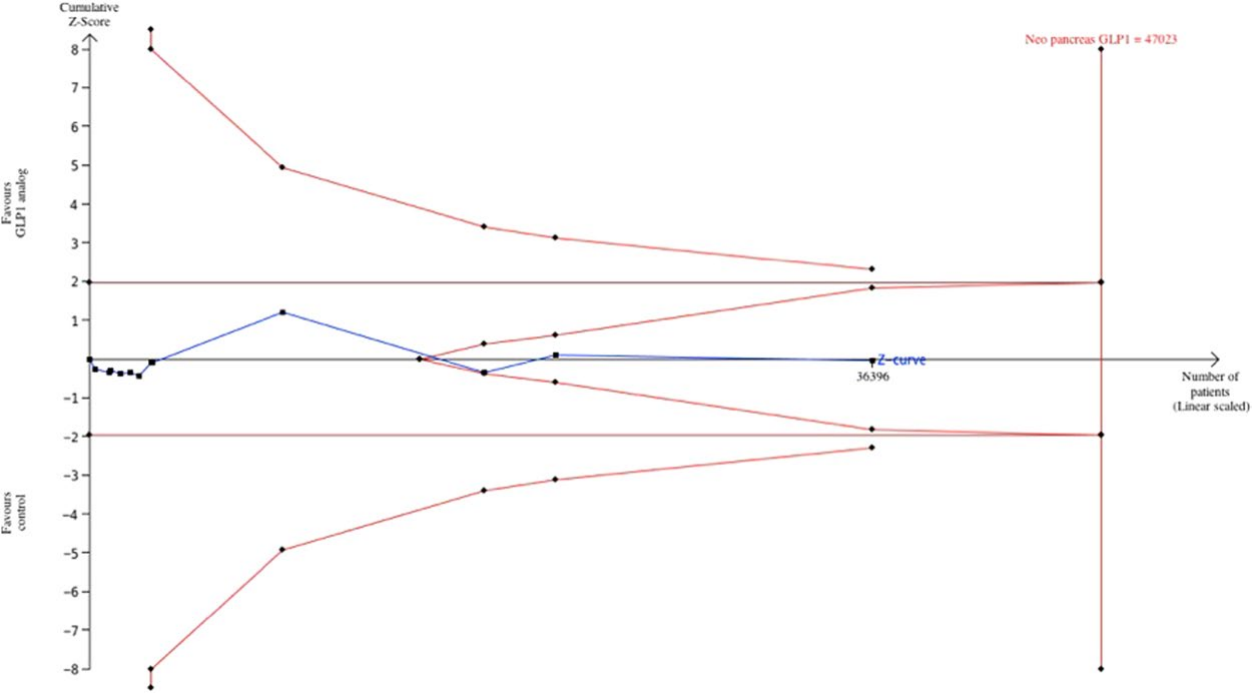
TSA for pancreatic cancer.

GLP-1 analogues did not increase the risk for pancreatic cancer when compared to the control OR 1.06; 95% CI 0.67 to 1.67; *I*^2^ 14%) (Fig. 2). When this analysis was repeated using only adjudicated trials, the results were left unchanged (OR 1.00; 95% CI 0.62 to 1.63; *I*^2^ 61%). TSA showed that the ideal sample size was 47,023, which was not reached (36,397). However, as the futility boundary was crossed, there is enough data to exclude the association between GLP-1 agonists treatment and pancreatic cancer (considering a difference of 0.1% between treatment groups) (Fig. 3). Considering results from all patients exposed to GLP-1 agonist use, the medication is safe, and a NNH as high as 1000 can be rejected. Funnel plot analysis did not show any small study bias (p = 0.721).

The LEADER trial reported pancreatic cancer incidence in more than one way. The first approach used only the adjudicated cases (GLP-1 n = 13 and placebo n = 5). This analysis is depicted in Fig. 2. Their second approach identified four additional cases of death, which were attributed to malignancy related to pancreatic cancer (but without histological documentation) by the adjudication committee (GLP-1 n = 13 and placebo n = 9). Repeating the meta-analysis with this additional information did not change the results (OR 0.95 95% CI 0.61 to 1.48; *I*^2^ 0%).

To investigate if trial duration influences the outcome, we performed a meta-regression. No association was found (p = 0.812; 95% CI −1.12 to 1.37) between trial duration and pancreatic cancer risk, but this analysis lacked power as only 7 studies were included in this analysis.

## Discussion

Our findings reinforce that GLP-1 analogue use is not associated with pancreatic cancer. This conclusion is based on randomized studies with a mean follow-up of 1.74 years (minimum 1 year – maximum 3.5 years) and confirmation through TSA that enough patients have been studied so far to exclude this association for this length of time, long-term associations cannot be analysed with the current studies.

As our findings are based on good quality randomized trials, confounding and attrition bias are controlled, and the risk of unreliable results is diminished. Most importantly, our meta-analysis adds evidence to previous meta-analyses^12,18,21^, as it is the only one to incorporate the TSA approach, which allowed us to exclude a clinically relevant magnitude of the association between GLP-1 analogues and pancreatic cancer. In other words, we achieved a number needed to harm (NNH) as high as 1537 patients.

We must acknowledge that our findings are based on studies with different follow-up durations and pancreatic cancer definitions. We explored these limitations with meta-regression, as well as with subgroup analysis, and the results were unchanged. In addition, in a search of clinicaltrials.gov, 87 ongoing trials with GLP-1 analogues were found. To reach a higher NNH, the results of these trials will need to be taken into account. Another point to be considered is the 17 trials with unreported pancreatic cancer events, from which we only received replies of 4 authors.

Compared to previous studies, these results are reassuring. There have been concerns regarding the safety of GLP-1 agonists since Raufman *et al*. reported in the early 90 s that GLP-1 interacted with exendin receptors on dispersed acini from guinea pig pancreas^22,23^, and these concerns were increased with the results from the LEADER trial showing a numerically greater, although statistically not significant, number of cases of pancreatic cancer in the liraglutide arm compared to the placebo^20^.

This study has some limitations. The follow up duration of the included trials may not be sufficient for the occurrence of carcinogenesis. We performed metarregression to evaluate if there was a trend towards higher incidence of pancreatic cancer in studies with longer duration, however no association between duration and the outcome was observed. Second, the limits of the confidence interval of the main outcome were 0.67 and 1.67, meaning that the real value of the statistics in 95% of the cases is somewhere in between those values, what indicates that besides the lack of association reported in this review, there is a chance that the medication could increase (as well as decrease) the risk of pancreatic cancer. In order to reassure our results, we performed direct meta-analysis and TSA, and in both cases the estimate was close to 1.0. Finally, it would be interesting to analyse the effect of each medication of the class separately, however due to the small number of studies with each one of them this analysis would lack power.

Our findings are relevant to patients with diabetes and obesity, as well as for physicians, as they reinforce the position of the European Medicines Agency, which considers incretins safe for use regarding pancreatic disease. In addition, other studies, most of which were observational, have shown similar results^24,25^.

Ultimately, our analysis did not find an association of GLP-1 analogue use with pancreatic cancer in a mena follow-up of 1.74 years, and a sufficient number of patients have been randomized to be able to exclude a NNH of more than 1000 patients. To exclude smaller risks (i.e., a larger NNH) and to aside concerns regarding the influence of longer duration of medication exposition and the outcome, further evidence is needed.

## Supplementary information

Supplementary Dataset 1
